# Complete genome sequence of *Granulicella mallensis* type strain MP5ACTX8^T^, an *acidobacterium* from tundra soil

**DOI:** 10.4056/sigs.4328031

**Published:** 2013-09-30

**Authors:** Suman R. Rawat, Minna K. Männistö, Valentin Starovoytov, Lynne Goodwin, Matt Nolan, Loren J. Hauser, Miriam Land, Karen Walston Davenport, Tanja Woyke, Max M. Häggblom

**Affiliations:** 1 Department of Biochemistry and Microbiology, Rutgers, The State University of New Jersey, New Brunswick, New Jersey USA; 2 Finnish Forest Research Institute, Rovaniemi, Finland; 3 Department of Cell Biology and Neuroscience, Rutgers, The State University of New Jersey, Piscataway, New Jersey, USA.; 4 Los Alamos National Laboratory, Bioscience Division, Los Alamos, New Mexico, USA; 5 DOE Joint Genome Institute, Walnut Creek, California, USA; 6 Oak Ridge National Laboratory, Oak Ridge, Tennessee, USA

**Keywords:** cold adapted, acidophile, tundra soil, *Acidobacteria*

## Abstract

*Granulicella mallensis* MP5ACTX8^T^ is a novel species of the genus *Granulicella* in subdivision 1of *Acidobacteria*. *G. mallensis* is of ecological interest being a member of the dominant soil bacterial community active at low temperatures and nutrient limiting conditions in Arctic alpine tundra. *G. mallensis* is a cold-adapted acidophile and a versatile heterotroph that hydrolyzes a suite of sugars and complex polysaccharides. Genome analysis revealed metabolic versatility with genes involved in metabolism and transport of carbohydrates. These include gene modules encoding the carbohydrate-active enzyme (CAZyme) family involved in breakdown, utilization and biosynthesis of diverse structural and storage polysaccharides including plant based carbon polymers. The genome of *Granulicella mallensis* MP5ACTX8^T^ consists of a single replicon of 6,237,577 base pairs (bp) with 4,907 protein-coding genes and 53 RNA genes.

## Introduction

Strain MP5ACTX8^T^ (= ATCC BAA-1857^T^ = DSM 23137^T^), is the type strain of the species *Granulicella mallensis* [1]. The genus *Granulicella,* in subdivision 1 of *Acidobacteria*, was first described by Pankratov *et al*. in 2010 [2]. *Granulicella mallensis* (mal.len' sis. N. L. fem. adj. *mallensis*; pertaining to its isolation from soil of Malla Nature Reserve, Kilpisjärvi, Finland; 69°01’N, 20°50’E) was described along with other species of the genus *Granulicella* isolated from tundra soil [1] and is one of the two with sequenced genomes, out of eight validly described *Granulicella* species.

*Acidobacteria* is one of the most ubiquitous bacterial phyla found in diverse habitats and is abundant in most soil environments [3,4] including Arctic tundra soils [5,6]. *Acidobacteria* are phylogenetically and physiologically diverse [7] represented by 26 phylogenetic subdivisions [8] of which only subdivisions 1, 3, 4, 8, and 10 are defined by taxonomically characterized representatives. To date, subdivision 1 is comprised of eight genera: *Acidobacterium* [9], *Terriglobus* [10,11], *Edaphobacter* [12], *Granulicella* [1,2], *Acidipila* [13], *Telmatobacter* [14], *Acidicapsa* [15] and *Bryocella* [16]. Subdivision 3, 4 and 10 include only one genus each, namely *Bryobacter* [17], *Blastocatella* [18] and *Thermotomaculum* [19], respectively, while subdivision 8 includes three genera; *Holophaga* [20], *Geothrix* [21] and *Acanthopleuribacter* [22]. Three species, ‘*Candidatus* Koribacter versatilis’ [23], ‘*Candidatus* Solibacter usitatus’ [23] and ‘*Candidatus* Chloracidobacterium thermophilum’ [24] have been described as ‘Candidatus’ taxa. *Acidobacteria* are relatively difficult to cultivate with slow growth rates and typically require up to several weeks to develop visible colonies on solid media. Nevertheless, the phylogenetic diversity, ubiquity and abundance of this group suggest that they play important ecological roles in soils. The abundance of *Acidobacteria* has been found to correlate with soil pH [25,26] and carbon [27,28], with subdivision 1 *Acidobacteria* being most abundant in slightly acidic soils. Our previous studies have shown that *Acidobacteria* dominate in the acidic tundra heaths of northern Finland [25,29-31]. Using selective isolation techniques we have been able to isolate several slow growing and fastidious strains of *Acidobacteria* [1,11]. On the basis of phylogenetic, phenotypic and chemotaxonomic data, including 16S rRNA, rpoB gene sequence similarity and DNA–DNA hybridization, strain MP5ACTX8^T^ was classified as a novel species of the genus *Granulicella* [1]. Here, we summarize the physiological features together with the complete genome sequence, annotation and data analysis of *Granulicella mallensis* MP5ACTX8^T^ (Table 1).

**Table 1:** Classification and general features of *G. mallensis* strain MP5ACTX8^T^ according to the MIGS recommendations [32]

**MIGS ID**	**Property**	**Term**	**Evidence code^a^**
	Classification	Domain *Bacteria*	TAS [33]
		Phylum *Acidobacteria*	TAS [34,35]
		Class *Acidobacteria*	TAS [36,37]
		Order *Acidobacteriales*	TAS [36,38]
		Family *Acidobacteriaceae*	TAS [34,39]
		Genus *Granulicella*	TAS [1,2]
		Species *Granulicella mallensis*	TAS [1]
		Type strain: MP5ACTX8^T^ (= ATCC BAA-1857^T^ = DSM 23137^T^)	
	Gram stain	negative	TAS [1]
	Cell shape	rod	TAS [1]
	Motility	non-motile	TAS [1]
	Sporulation	not reported	NAS
	Temperature range	4–28 °C	TAS [1]
	Optimum temperature	24–27 °C	TAS [1]
	pH range	3.5–6.5	TAS [1]
	Optimum pH	5	TAS [1]
			
			
	Carbon source	D-glucose, maltose, D-fructose, D-galactose, lactose, lactulose, D-mannose, D-ribose, raffinose, sucrose, trehalose, cellobiose, D-xylose, glucuronate	TAS [1]
MIGS-6	Habitat	terrestrial	TAS [1]
MIGS-6.3	Salinity	Growth with up to 1.5% NaCl	TAS [1]
MIGS-22	Oxygen requirement	aerobic	TAS [1]
MIGS-15	Biotic relationship	free-living	TAS [1]
MIGS-14	Pathogenicity	non-pathogenic	NAS
MIGS-4	Geographic location	Arctic-alpine tundra, Finland	TAS [1]
MIGS-5	Sample collection	2006	TAS [1]
MIGS-4.1	Latitude	69°01’N,	TAS [1]
MIGS-4.2	Longitude	20°50’E	
MIGS-4.4	Altitude	700 m	TAS [1]

^a^Evidence codes - IDA: Inferred from Direct Assay; TAS: Traceable Author Statement (i.e., a direct report exists in the literature); NAS: Non-traceable Author Statement (i.e., not directly observed for the living, isolated sample, but based on a generally accepted property for the species, or anecdotal evidence). These evidence codes are from the Gene Ontology project [40].

## Classification and features

**Figure 1:**
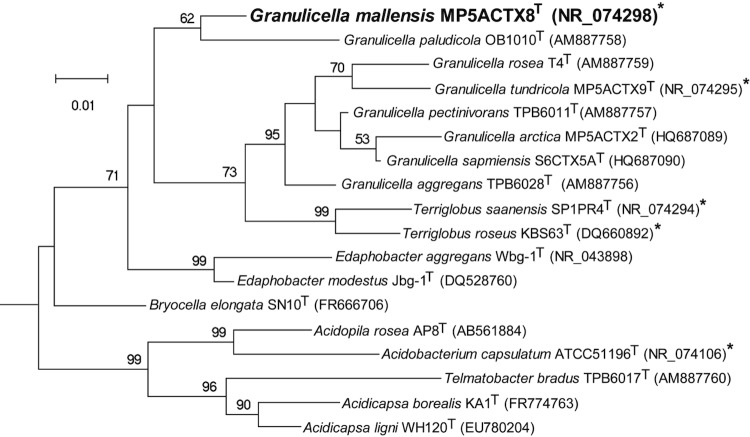
Phylogenetic tree highlighting the position of *G. mallensis* MP5ACTX8^T^ (shown in bold) relative to the other type strains within SD1 *Acidobacteria*. The maximum likelihood tree was inferred from 1,361 aligned positions of the 16S rRNA gene sequences and derived based on the Tamura-Nei model using MEGA 5 [41]. Bootstrap values >50 (expressed as percentages of 1,000 replicates) are shown at branch points. Bar: 0.02 substitutions per nucleotide position. The corresponding GenBank accession numbers are displayed in parentheses. Strains whose genomes have been sequenced, are marked with an asterisk; *G. mallensis* MP5ACTX8^T^ (CP003130), *G. tundricola* MP5ACTX9^T^ (CP002480), *T. saanensis* SP1PR4^T^ (CP002467), *T. roseus* KBS63^T^ (CP003379) and *A. capsulatum* ATCC 51196^T^ (CP001472). *Bryobacter aggregatus* MPL3 (AM162405) in SD3 *Acidobacteria* was used as an outgroup.

Within the genus *Granulicella*, eight species are described with validly published names: *G. mallensis* MP5ACTX8^T^, *G. tundricola* MP5ACTX9^T^, *G. arctica* MP5ACTX2^T^ and *G. sapmiensis* S6CTX5A^T^ isolated from Arctic tundra soil [1] and *G. paludicola* OB1010^T^, *G. pectinivorans* TPB6011^T^, *G. rosea* TPO1014^T^ and *G. aggregans* TPB6028^T^ isolated from sphagnum peat bogs [3]. Strain MP5ACTX8^T^ showed 95.5 -96.1% 16S rRNA gene sequence identity to tundra soil strains, *G. tundricola* MP5ACTX9^T^ (95.5%), *G. sapmiensis* S6CTX5A^T^ (96.2%) and *G. arctica* MP5ACTX2^T^ (96.1%) and 94.6 – 97.4% to *G. rosea* TPO1014^T^ (94.6%), *G. aggregans* TPB6028^T^ (96.0%), *G. pectinivorans* TPB6011^T^ (96.1%), *G. paludicola* OB1010^T^ (96.5%) and *G. paludicola* LCBR1 (97.4%). Phylogenetic analysis based on the 16S rRNA gene of taxonomically classified strains of family *Acidobacteriaceae* placed *G. paludicola* type strain OB1010 ^T^ as the closest taxonomically classified relative of *G. mallensis* MP5ACTX8^T^ (Figure 1).

### Morphology and physiology

**Figure 2:**
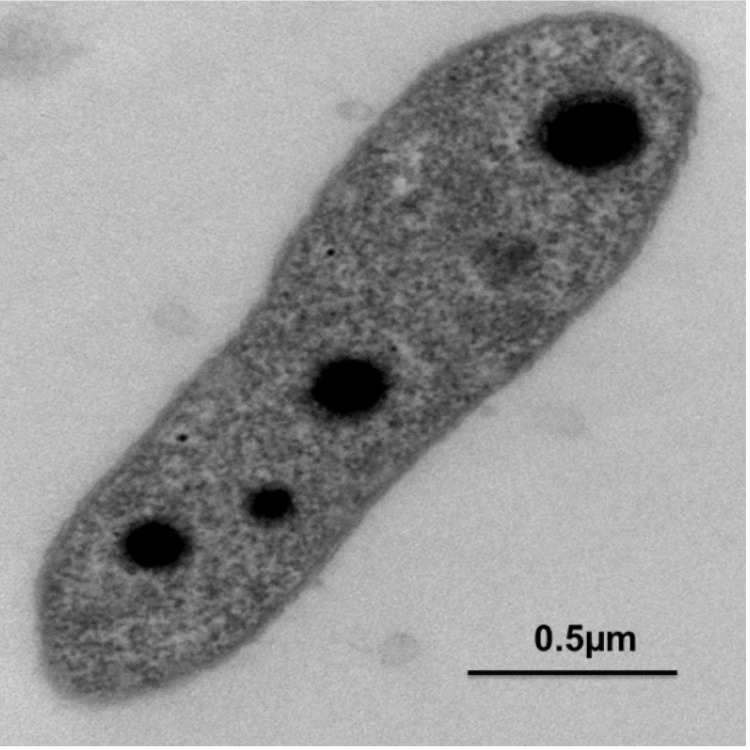
Electron micrograph of *G. mallensis* MP5ACTX8^T^.

*G. mallensis* grows on R2 medium (Difco) at pH 3.5–6.5 (optimum pH 5) and at +4 to +28 °C (optimum 24–27 °C) [1]. On R2 agar, strain MP5ACTX8^T^ forms opaque white mucoid colonies with a diameter of approximately 1 mm. Cells are Gram-negative, non-motile, aerobic rods, approximately 0.5–0.7 mm wide and 0.6–1.3 mm long. Growth observed with up to 1.5% NaCl (w/v) (Table 1). The cell-wall structure in ultrathin sections of electron micrographs of cells of MP5ACTX8^T^ is shown in Figure 2.

*G. mallensis* utilizes D-glucose, maltose, cellobiose, D-fructose, D-galactose, lactose, lactulose, D-mannose, D-ribose, raffinose, sucrose, trehalose, D-xylose, N-acetyl-D-glucosamine, glucuronate, glutamate, melezitose and salicin, but does not utilize D-arabinose, acetate, formate, pyruvate, malate, mannitol, D- or L-alanine, D-glycine, L-leucine, L-ornithine, gluconic acid, aspartate, dulcitol, butyrate, caproate, valerate, lactate, oxalate, propionate, fumarate, adonitol, methanol, ethanol, succinate, D-sorbitol or myoinositol, when grown using VL55 mineral medium with 100 mg yeast extract l^-1^. *G. mallensis* hydrolyzes aesculin, starch, pectin, laminarin and lichenan, but not gelatin, cellulose, xylan, sodium alginate, pullulan, chitosan or chitin on R2 medium. Strains show positive reaction for acid and alkaline phosphatases, leucine arylamidase, a-chymotrypsin, naphthol-AS-BI-phosphohydrolase, α- and β-galactosidases, α- and β-glucosidases, N-acetyl- β-glucosaminidase, β-glucuronidase, trypsin and valine arylamidase, but negative for α-fucosidase, α-mannosidase, esterase (C4 and C8), lipase (C14) and cystine arylamidase. Strain MP5ACTX8^T^ reduces nitrate to nitrite. Strain MP5ACTX8^T^ is resistant to the antibiotics erythromycin, chloramphenicol, neomycin, rifampicin, streptomycin, gentamicin, polymyxin B and penicillin, but susceptible to ampicillin, kanamycin, tetracycline, lincomycin, novobiocin and bacitracin [1].

### Chemotaxonomy

The major cellular fatty acids in *G. mallensis* are iso-C_15:0_ (45.3%), C_16:1ω7c_ (28.7%), iso-C_13:0_ (8.3%) and C_16:0_ (8.9%). The cellular fatty acid compositions of strain MP5ACTX8^T^ were relatively similar to that of other *Granulicella* strains with fatty acids iso-C_15:0_ and C_16:1ω7c_ being most abundant in all strains. Strain MP5ACTX8^T^ contains MK-8 as the major quinone.

## Genome sequencing and annotation

### Genome project history

*G. mallensis* strain MP5ACTX8^T^ was selected for sequencing in 2009 by the DOE Joint Genome Institute (JGI) community sequencing program. The Quality Draft (QD) assembly and annotation were completed on December 26, 2010. The complete genome was made available on Dec. 1, 2011. The genome project is deposited in the Genomes On-Line Database (GOLD) [42] and the complete genome sequence of strain MP5ACTX8^T^ is deposited in GenBank (CP003130). Table 2 presents the project information and its association with MIGS version 2.0 [32].

**Table 2:** Project information.

**MIGS ID**	**Property**	**Term**
MIGS 31	Finishing quality	Finished
MIGS-28	Libraries used	Three libraries, an Illumina GAii shotgun library (GSGY), a 454 Titanium standard library (GSXT, GWTA) and a paired end 454 (GSFP) library
MIGS 29	Sequencing platforms	454 Titanium standard, 454 Paired End, Illumina
MIGS 31.2	Fold coverage	18.5× (454), 213× (Illumina)
MIGS 30	Assemblers	Newbler, VELVET, PHRAP
MIGS 32	Gene calling method	ProdigaL, GenePRIMP
	Locus Tag	AciX8
	Genbank ID	CP003130.1
	GenBank Date of Release	December 1, 2011
	GOLD ID	Gc02349
	BIOPROJECT	PRJNA49957, PRJNA47903
	Project relevance	Environmental, Biogeochemical cycling of Carbon, Biotechnological, GEBA

### Growth conditions and genomic DNA extraction

*G. mallensis* MP5ACTX8^T^ was cultivated on R2 medium as previously described [1]. Genomic DNA (gDNA) of high sequencing quality was isolated using a modified CTAB method and evaluated according to the Quality Control (QC) guidelines provided by the DOE Joint Genome Institute [43].

### Genome sequencing and assembly

The finished genome of *G. mallensis* MP5ACTX8^T^ (JGI ID 4088692) was generated at the DOE Joint genome Institute (JGI) using a combination of Illumina [44] and 454 technologies [45]. For this genome, an Illumina GAii shotgun library which generated 59,701,420 reads totaling 4537.3 Mb, a 454 Titanium standard library which generated 136,708 reads and a paired end 454 library with an average insert size of 10.3 kb which generated 157,336 reads totaling 172.0 Mb of 454 data, were constructed and sequenced. All general aspects of library construction and sequencing performed at the JGI can be found at the JGI website [43]. The 454 Titanium standard data and the 454 paired end data were assembled with Newbler, version 2.3. Illumina sequencing data was assembled with Velvet, version 0.7.63 [46]. The 454 Newbler consensus shreds, the Illumina Velvet consensus shreds and the read pairs in the 454 paired end library were integrated using parallel phrap, version SPS - 4.24 (High Performance Software, LLC) [47]. The software Consed [48] was used in the finishing process. The Phred/Phrap/Consed software package [49] was used for sequence assembly and quality assessment in the subsequent finishing process. Illumina data was used to correct potential base errors and increase consensus quality using the software Polisher developed at JGI (Alla Lapidus, unpublished). Possible misassemblies were corrected using gapResolution (Cliff Han, un-published), Dupfinisher [50] or sequencing cloned bridging PCR fragments with sub-cloning. Gaps between contigs were closed by editing in Consed, by PCR and by Bubble PCR (J-F Cheng, unpublished) primer walks. The final assembly is based on 74.2 Mb of 454 data which provides an average 18.5× coverage and 1318.5 Mb of Illumina data which provides an average 213× coverage of the genome.

### Genome annotation

Genes were identified using Prodigal [51] as part of the Oak Ridge National Laboratory genome annotation pipeline, followed by a round of manual curation using the JGI GenePRIMP pipeline [52]. The predicted CDSs were translated and used to search the National Center for Biotechnology Information (NCBI) non-redundant database, UniProt, TIGRFam, Pfam, PRIAM, KEGG, COGs [53,54], and InterPro. These data sources were combined to assert a product description for each predicted protein. Non-coding genes and miscellaneous features were predicted using tRNAscan-SE [55], RNAMMer [56], Rfam [57], TMHMM [58], and signalP [59]. Additional gene prediction analysis and functional annotation were performed within the Integrated Microbial Genomes Expert Review (IMG-ER) platform [60].

## Genome properties

**Figure 3:**
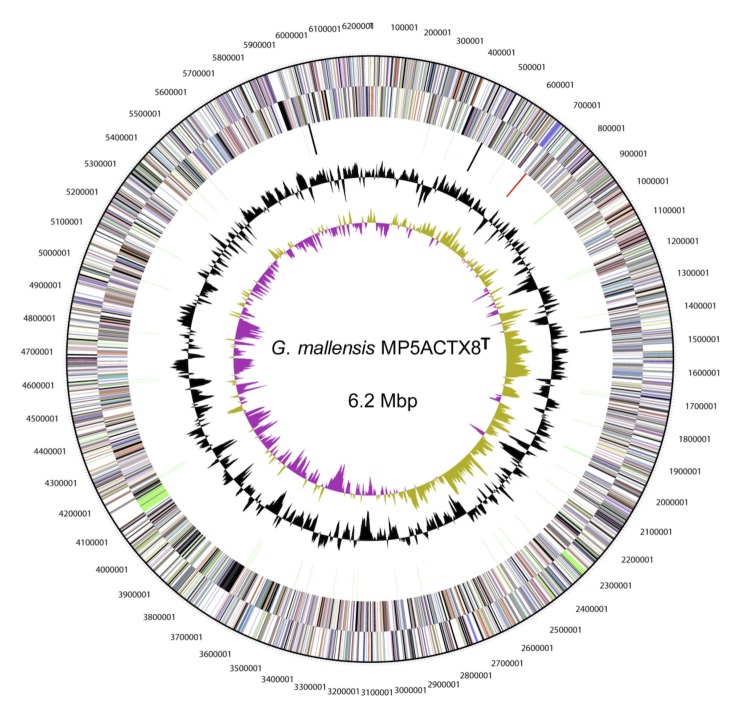
Circular representation of the chromosome of *G. mallensis* MP5ACTX8^T^ displaying relevant genome features. From outside to center; Genes on forward strand (color by COG categories), genes on reverse strand (color by COG categories), RNA genes (tRNAs green, rRNAs red, other RNAs black), GC content and GC skew.

The genome consists of one circular chromosome of 6,211,694 bp in size with a GC content of 57.8 mol% and consists of 53 RNA genes (Figure 3 and Table 3). Of the 4,960 predicted genes, 4,907 are protein-coding genes (CDSs) and 90 are pseudogenes. Of the total CDSs, 70.5% represent COG functional categories and 16% consist of signal peptides. The distribution of genes into COG functional categories is presented in Figure 3 and Table 4.

**Table 3:** Genome statistics

**Attribute**	**Value**	**% of Total**
Genome size (bp)	6,237,577	100
DNA coding region(bp)	5,499,388	88.2
DNA G+C content (bp)	3612173	57.9
DNA scaffolds	1	100
Total genes	4,960	100
Protein coding genes	4,907	98.9
RNA genes	53	1.3
Pseudo genes	90	1.8
Genes in internal clusters	2,679	54
Genes with function prediction	3,511	70.8
Genes assigned to COGs	3,496	70.5
Genes with Pfam domains	3,754	75.7
Genes with signal peptides	797	16.1
Genes with transmembrane helices	1,291	26.0
CRISPR repeats	0	-

The total is based on either the size of the genome in base pairs or the protein coding genes in the annotated genome.

**Table 4:** Number of genes associated with general COG functional categories

**Code**	**Value**	**%age**	**Description**
J	167	4.32	Translation, ribosomal structure and biogenesis
A	2	0.05	RNA processing and modification
K	332	8.58	Transcription
L	156	4.03	Replication, recombination and repair
B	1	0.03	Chromatin structure and dynamics
D	27	0.7	Cell cycle control, Cell division, chromosome partitioning
Y	0.0	0.0	Nuclear structure
V	76	1.96	Defense mechanisms
T	139	3.59	Signal transduction mechanisms
M	322	8.32	Cell wall/membrane biogenesis
N	17	0.44	Cell motility
Z	0.0	0.0	Cytoskeleton
W	0.0	0.0	Extracellular structures
U	79	2.04	Intracellular trafficking and secretion
O	123	3.18	Posttranslational modification, protein turnover, chaperones
C	193	4.99	Energy production and conversion
G	355	9.18	Carbohydrate transport and metabolism
E	258	6.67	Amino acid transport and metabolism
F	76	1.96	Nucleotide transport and metabolism
H	155	4.01	Coenzyme transport and metabolism
I	164	4.24	Lipid transport and metabolism
P	157	4.06	Inorganic ion transport and metabolism
Q	125	3.23	Secondary metabolites biosynthesis, transport and catabolism
R	527	13.62	General function prediction only
S	418	10.8	Function unknown
-	1,464	29.52	Not in COGs

The total is based on the total number of protein coding genes in the genome.

## Discussion

*Granulicella mallensis* type strain MP5ACTX8^T^ has the largest genome size of 6.2 Mbp. among the three tundra soil strains of subdivision 1 *Acidobacteria* [28]. Genome analysis of *Granulicella mallensis* identified a high abundance of genes assigned to COG functional categories for transport and metabolism of carbohydrates (9.1%) and amino acids (6.7%) and involved in cell envelope biogenesis (8.3%) and transcription (8.6%). Further genome analysis revealed an abundance of gene modules encoding for functional activities within the carbohydrate-active enzymes (CAZy) family [61] involved in breakdown, utilization and biosynthesis of carbohydrates. *G. mallensis* hydrolyzed complex carbon polymers, including CMC, pectin, lichenin, laminarin and starch, and utilized sugars such as cellobiose, D-mannose, D-xylose, D-trehalose. This parallels genome predictions for CDSs encoding for enzymes such as cellulases, pectinases, alginate lyases, trehalase and amylases. In addition, the *G. mallensis* genome contained a cluster of genes in the neighborhood of the cellulose synthase gene (bcsAB) which included cellulase (bscZ) (endoglucanase Y) of family GH8, cellulose synthase operon protein (bcsC) and a cellulose synthase operon protein (yhjQ) involved in cellulose biosynthesis. Detailed comparative genome analysis of *G. mallensis* MP5ACTX8^T^ with other *Acidobacteria* strains for which finished genomes were available is reported in Rawat et al. [28]. The data thus suggests that *G. mallensis* is involved in hydrolysis, the utilization of stored carbohydrates, and in the biosynthesis of exopolysaccharides from organic matter and plant based polymers in the soil. Therefore, we infer that strain *G. mallensis* may be central to carbon cycling processes in arctic and boreal soil ecosystems.
